# Author's reply

**DOI:** 10.1192/bjb.2022.13

**Published:** 2022-04

**Authors:** Emmeline Lagunes-Cordoba

**Affiliations:** Specialty Doctor, Camden and Islington NHS Foundation Trust, UK. Email: mcanoline@hotmail.com

We agree with the comment regarding how ethnic minority is an independent effect with respect to differential attainment; however, this paper tried to focus on the subgroup showing the largest effect size, IMGs.

The comment regarding IMGs of White ethnicity is an illustration of the above (ethnicity: moderate effect on differential attainment; ‘IMG-ness’: large effect on differential attainment), so we are grateful you have helped us to make this point more explicit. However, we think the central point of our paper remains – that IMGs need special focus as, unlike ethnicity, to be an IMG is not a protected characteristic, so interventions to support IMG might not be deemed to be a priority or even a need. Overall, we consider that this is complex and delicate, with many further layers of intersectionality, including gender, sexual orientation and social background, but it is promising that more and more work, including yours, is beginning to address the many issues affecting IMGs working in the UK.

